# A longitudinal study of topic classification on Twitter

**DOI:** 10.7717/peerj-cs.991

**Published:** 2022-06-07

**Authors:** Mohamed Reda Bouadjenek, Scott Sanner, Zahra Iman, Lexing Xie, Daniel Xiaoliang Shi

**Affiliations:** 1 School of Information Technology, Deakin University, Geelong, Victoria, Australia; 2 Department of Mechanical and Industrial Engineering, University of Toronto, Toronto, Ontario, Canada; 3 Computer Science, Oregon State University, Corvalis, Oregon, United States; 4 Computer Science, Australian National University, Canberra, ACT, Australia

**Keywords:** Social network analysis, Topic classification, Data analysis

## Abstract

Twitter represents a massively distributed information source over topics ranging from social and political events to entertainment and sports news. While recent work has suggested this content can be narrowed down to the personalized interests of individual users by training topic filters using standard classifiers, there remain many open questions about the efficacy of such classification-based filtering approaches. For example, over a year or more after training, how well do such classifiers generalize to future novel topical content, and are such results stable across a range of topics? In addition, how robust is a topic classifier over the time horizon, *e.g*., can a model trained in 1 year be used for making predictions in the subsequent year? Furthermore, what features, feature classes, and feature attributes are most critical for long-term classifier performance? To answer these questions, we collected a *corpus* of over 800 million English Tweets *via* the Twitter streaming API during 2013 and 2014 and learned topic classifiers for 10 diverse themes ranging from social issues to celebrity deaths to the “Iran nuclear deal”. The results of this long-term study of topic classifier performance provide a number of important insights, among them that: (i) such classifiers can indeed generalize to novel topical content with high precision over a year or more after training though performance degrades with time, (ii) the classes of hashtags and simple terms contain the most informative feature instances, (iii) removing tweets containing training hashtags from the validation set allows better generalization, and (iv) the simple volume of tweets by a user correlates more with their informativeness than their follower or friend count. In summary, this work provides a long-term study of topic classifiers on Twitter that further justifies classification-based topical filtering approaches while providing detailed insight into the feature properties most critical for topic classifier performance.

## Introduction

With the emergence of the Social Web in the mid-2000s, the Web has evolved from a static Web, where users were only able to consume information, to a Web where users are also able to interact and produce information (Bouadjenek, Hacid & Bouzeghoub, 2016). This evolution, which is commonly known as the Social Web, has introduced new freedoms for the user in their relation with the Web by facilitating their interactions with other users who have similar tastes or share similar resources. Specifically, social media platforms such as Twitter are commonly used as a means to communicate with other users and to post messages that express opinions and topics of interest. In 2019, it was estimated that more than 330 million users posted 500 million tweets per day (https://www.brandwatch.com/blog/twitter-stats-and-statistics/).

Consequently, Twitter represents a double-edged sword for users. On one hand it contains a vast amount of novel and topical content that challenge traditional news media sources in terms of their timeliness and diversity. Yet on the other hand Twitter also contains a vast amount of chatter and otherwise low-value content for most users’ information needs where manual filtering of irrelevant content can be extremely time-consuming. Previous work by Lin, Snow & Morgan (2011), Yang et al. (2014) and Magdy & Elsayed (2014) has noted the need for topic-based filtering on Twitter and has proposed a range of variations on supervised classification techniques to build effective topic filters.

While these previous approaches have augmented their respective topical classifiers with extensions including semi-supervised training of multiple stages of classification-based filtering and online tracking of foreground and background language model evolution, we seek to analyze the lowest common denominator of all of these methods, namely the performance of the underlying (vanilla) supervised classification paradigm. Our fundamental research questions in this article are hence focused on a longitudinal study of the performance of such supervised topic classifiers. For example, over a year or more after training, how well do such classifiers generalize to future novel topical content, and are such results stable across a range of topics? In addition, how robust is a topic classifier over the time horizon, *e.g*., can a model trained in 1 year be used for making predictions in the subsequent year? Furthermore, what features, feature classes, and feature attributes are most critical for long-term classifier performance?

To answer these questions, we collected a *corpus* of over 800 million English Tweets *via* the Twitter streaming API during 2013 and 2014 and learned topic classifiers for 10 diverse themes ranging from social issues to celebrity deaths to the “Iran nuclear deal”. We leverage ideas from Lin, Snow & Morgan (2011) for curating hashtags to define our 10 training topics and label tweets for supervised training; however, we also curate disjoint hashtag sets for validation and test data to tune hyperparameters and evaluate true generalization performance of the topic filters to future novel content.

The main outcomes of this work can be summarized as follows:

- We empirically show that the random forest classifier generalizes well to unseen future topical content (including content with no hashtags) in terms of its average precision (AP) and Precision@*n* (for a range of *n*) evaluated over long time-spans of typically 1 year or more after training.
- We demonstrate that the performance of classifiers tends to drop over time–roughly 35% drop in Mean Average Precision 350 days after training ends, which is an expected, but nonetheless significant decrease. We attribute this to the fact that over long periods of time, features that are predictive during the training period may prove ephemeral and fail to generalize to prediction at future times.
- To address the problem above, we show that one can remove tweets containing training hashtags from the validation set to allow better parameter tuning leading to less overfitting and improved long-term generalization. Indeed, although our approach here is simple, it yields a roughly 11% improvement for Mean Average Precision.
- Finally, we provide a detailed analysis of features and feature classes and how they contribute to classifier performance. Among numerous insights, we show that the class of hashtags and simple terms have some of the most informative feature instances. We also show that the volume of tweets by a user correlates more with their informativeness than their follower or friend count.

In summary, this work^1^ 1This is an extended and revised version of a preliminary conference report that was presented in Iman et al. (2017). provides a longitudinal study of Twitter topic classifiers that further justifies supervised approaches used in existing work while providing detailed insight into feature properties and training methodologies leading to good performance. The rest of this article is organized as follows: we first review the literature and then describe the notation we use in this article as well as a formal definition of the problem we address. Next, we provide a description of the dataset we used for the analysis in this article, followed by a description of the general methodology we use for learning topic classifiers. Finally, we provide a discussion of our empirical results before concluding and outlining future work.

## Related work

There is a substantial body of research related to topic classification in social media. Below, we review the major works related to Twitter topic classification, topic modeling for social media and applications of classifiers for social media (including tweet recommendation, event detection in social media, and “friend sensors”).

### Twitter topic classification

Topic classification for social media aims to detect and track general topics such as “Baseball” or “Fashion”. In previous work, researchers have collected labeled data either by using a single hashtag for each topic (Lin, Snow & Morgan, 2011), a user-defined query for each topic (Magdy & Elsayed, 2014), manual labeling (Daouadi, Zghal Rebaï & Amous, 2021; Ayo et al., 2021), or co-training based on the URLs and text of the tweet (Yang et al., 2014). We expand on Lin, Snow & Morgan (2011)’s work and use a set of hashtags instead of a single hashtag. Similarly, we extract features consisting of hashtags, mentions, unigram terms, and authors as done in this prior work, but also add location as another feature, which has shown to be the second most important feature for topic classification after unigram terms. Furthermore, we provided a novel learning and evaluation paradigm based on splitting both the data and hashtags along temporal boundaries to generate train, validation and test datasets in order to evaluate long-term generalization of trained topic classifiers. In contrast, we remark that Lin, Snow & Morgan (2011) only evaluated over 1 week, Magdy & Elsayed (2014) over 4 days, and Yang et al. (2014) did not explicitly mention the data duration or that their study was intended to assess long-term performance. Hence these previous studies do not permit one to assess the long-term topic classification performance of topic classifiers for Twitter as intended by the 2 year longitudinal study performed in this article.

### Topic modeling for social media

Topic models are a type of statistical model for discovering abstract “topics” that occur in a collection of documents (Blei, 2012). For this purpose, machine learning researchers have developed a suite of algorithms including Probabilistic Latent Semantic Analysis (PLSA) (Hofmann, 1999), Non-negative matrix factorization (Lee & Seung, 1999; Arora, Ge & Moitra, 2012; Luo et al., 2017), and Latent Dirichlet allocation (LDA) (Blei, Ng & Jordan, 2003). LDA is perhaps the most common topic model currently in use.

While topic models such as LDA have a long history of successful application to content domains such as news articles (Chen et al., 2010; Cohen & Ruths, 2013; Greene & Cross, 2015) and medical science (Paul & Dredze, 2011; Wu et al., 2012; Zhang et al., 2017), they are often less coherent when applied to social media and specifically microblog content like Twitter. In particular, Twitter poses challenges for topic modeling mainly because it contains short and messy text (Zhao et al., 2011b; Han, Cook & Baldwin, 2012; Mehrotra et al., 2013; Jelodar et al., 2018; Zuo et al., 2021). This problem has been frequently addressed through content pooling methods (Hong & Davison, 2010; Weng et al., 2010; Naveed et al., 2011; Mehrotra et al., 2013; Alvarez-Melis & Saveski, 2016), which comprise a data preprocessing step consisting of merging related tweets together and presenting them as a single document to the topic modeling algorithm. In a different vein, several works proposed to integrate network structure with topic modeling (Tang et al., 2008; Chen, Zhou & Carin, 2012b; Kim et al., 2012; Chen et al., 2017). Very recent work by Nolasco and Oliveira (Nolasco & Oliveira, 2019) proposed a method for detecting subevents within main complex events through topic modeling in social media posts.

Despite this rich tradition of work in topic modeling including applications to Twitter, we remark that all of these methods are unsupervised and seek to discover topics, whereas our work is focused on the supervised setting where topics (and their labels) are available and we are concerned with long-term classifier accuracy in this supervised, known topic setting.

### Related applications of classifiers for social media

Aside from highly related work on supervised topic classifiers for Twitter (Lin, Snow & Morgan, 2011; Yang et al., 2014; Magdy & Elsayed, 2014) that motivated this study as discussed previously, there are many other uses of classifiers for social media. While we argue no prior work has performed a longitudinal analysis of supervised Twitter topical classifiers as done in this article, these alternative applications of classifiers for social media may broadly benefit from the insights gained by our present study. We cover these related uses below along with important differences with the present work, divided into the following four subareas: (1) trending topic detection, (2) tweet recommendation, (3) friend sensors, and (4) specific event detection such as earthquake or influenza sensors.

**Trending Topic Detection** represents one of the most popular types of topical tweet detector and can be subdivided into many categories. The first general category of methods define trends as topically coherent content and focus on clustering across lexical, linguistic, temporal and/or spatial dimensions (Petrović, Osborne & Lavrenko, 2010; Ishikawa et al., 2012; Phuvipadawat & Murata, 2010; Becker, Naaman & Gravano, 2011; O’Connor, Krieger & Ahn, 2010; Weng & Lee, 2011). The second general category of methods define trends as temporally coherent patterns of terms or keywords and focus largely on detecting bursts of terms or phrases (Mathioudakis & Koudas, 2010; Cui et al., 2012; Zhao et al., 2011a; Nichols, Mahmud & Drews, 2012; Aiello et al., 2013). The third category of methods extends the previous categories by additionally exploiting network structure properties (Budak, Agrawal & El Abbadi, 2011). Despite this important and very active area of work that can be considered a type of topical tweet detector, trending topic detection is intrinsically unsupervised and not intended to detect targeted topics. In contrast, the work in this article is based on supervised learning of a specific topical tweet detector trained on the topical set of hashtags provided by the user.

**Tweet Recommendation** represents an alternate use of tweet classification and falls into two broad categories: personalized or content-oriented recommendation and retweet recommendation. For the first category, the objective of personalized recommendation is to observe a user’s interests and behavior from their user profile, sharing or retweet preferences, and social relations to generate tweets the user may like (Yan, Lapata & Li, 2012; Chen et al., 2012a). The objective of content-oriented recommendation is to use source content (*e.g*., a news article) to identify and recommend relevant tweets (*e.g*., to allow someone to track discussion of a news article) (Krestel et al., 2015). For the second category, there has been a variety of work on retweet prediction that leverages retweet history in combination with tweet-based, author-based, and social network features to predict whether a user will retweet a given tweet (Can, Oktay & Manmatha, 2013; Xu & Yang, 2012; Petrovic, Osborne & Lavrenko, 2011; Gilabert & Segu, 2021). Despite the fact that all of these methods recommend tweets, they—and recommendation methods in general—are not focused on a specific topic but rather on predicting tweets that correlate with the preferences of a specific user or that are directly related to specific content. Rather the focus with learning topical classifiers is to learn to predict for a broad theme (independent of a user’s profile) in a way that generalizes beyond existing labeled topical content to novel future topical content.

**Specific Event Detection** builds topical tweet detectors as we do in this work but focuses on highly specific events such as disasters or epidemics. For the use case of earthquake detection, an SVM can be trained to detect earthquake events and coupled with a Kalman filter for localization (Sakaki, Okazaki & Matsuo, 2013), whereas in Bouadjenek, Sanner & Du (2020), Bouadjenek & Sanner (2019) a relevance-driven clustering algorithm to detect natural disasters has been proposed. In another example use case to detect health epidemics such as influenza, researchers build purpose-specific classifiers targeted to this specific epidemic (Culotta, 2010; Aramaki, Maskawa & Morita, 2011), *e.g*., by exploiting knowledge of users’ proximity and friendship along with the contageous nature of influenza (Sadilek, Kautz & Silenzio, 2012). While these targeted event detectors have the potential of providing high precision event detection, they are highly specific to the target event and do not easily generalize to learn arbitrary topic-based classifiers for Twitter as analyzed in this work.

**Friend Sensors** are a fourth and final class of social sensors intended for early event detection (Kryvasheyeu et al., 2015; Garcia-Herranz et al., 2014) by leveraging the concept of the “friendship paradox” (Feld, 1991), to build user-centric social sensors. We note that our topical classifiers represent a *superset* of friend sensors since our work includes author features that the predictor may learn to use if this proves effective for prediction. However, as shown in our feature analysis, user-based features are among the least informative feature types for our topical classifier suggesting that general topical classifiers can benefit from a wide variety of features well beyond those of author features alone.

## Notation and problem definition

Our objective in this article is to carry out a longitudinal study of topic classifiers for Twitter. For each Twitter topic, we seek to build a binary classifier that can label a previously unseen tweet as topical (or not). To achieve this, we train and evaluate the classifier on a set of topically labeled historical tweets as described later in this article.

Formally, given an arbitrary tweet *d* (a document in text classification parlance) and a set of topics *T* = {*t*_1_,…,*t*_*K*_}, we wish to train *f*^*t*^(*d*) to predict a continuous score value for each topic *t* ∈ *T* over a subset of labeled training tweets from *D* = {*d*_1_,…,*d*_*N*_ }. We assume that each tweet *d*_*i*_ ∈ *D* (for *i* ∈ {1,…,*N* }) is represented by a vector of *M* binary features ${d_i} = [d_i^1, \ldots ,d_i^M]$ with $d_i^m \in \{ 0,1\}$ (for *m* ∈ {1,…,*M* }) indicating that the *m*th feature occurs in *d*_*i*_ (1) or not (0). Each tweet *d*_*i*_ also has an associated topic label $t({d_i}) \in \{ 0,1\}$ to indicate whether the tweet *d*_*i*_ is topical (1) or not (0). As done in many standard classifiers (*e.g*., naïve Bayes, logistic regression, SVM), we wish to learn a scoring function *f*^*t*^(*d*) such that a higher score *f*^*t*^(*d*) indicates a higher confidence that *d* should classified as topical for *t* and furthermore this generalizes well to new unseen tweet data not encountered during training.

## Data description

We begin with details of the Twitter testbed for topical classifier learning that we evaluate in this article. We crawled Twitter data using Twitter Streaming API for 2 years spanning 2013 and 2014 years. We collected more than 2.5 TB of compressed data, which contains a total number of 811,683,028 English tweets. In the context of Twitter, we consider five feature types for each tweet. Each tweet has a *User* feature (*i.e*., the person who tweeted it), a possible *Location* (*i.e*., a string provided as meta-data), and a time stamp when it was posted. A tweet can also contain one or more of the following:

- *Hashtag*: a topical keyword specified using the # sign.
- *Mention*: a Twitter username reference using the @ sign.
- *Term*: any non-hashtag and non-mention unigrams.

We provide more detailed statistics about each feature in Table 1. For example, there are over 11 million unique hashtags, the most frequent unique hashtag occurred in over 1.6 million tweets, a hashtag has been used on average by 10.08 unique users, and authors (*Users*) have used a median value of 2 tweets.

**Table 1:** Feature Statistics of our 811,683,028 tweet *corpus*.

**#Unique features**				
**User**	**Hashtag**	**Mention**	**Location**	**Term**
85,794,831	13,607,023	46,391,269	18,244,772	16,212,640
**Feature usage in #Tweets**				
**Feature**	**Max**	**Avg**	**Median**	**Most frequent**
**User**	10,196	8.67	2	running_status
**Hashtag**	1,653,159	13.91	1	#retweet
**Mention**	6,291	1.26	1	tweet_all_time
**Location**	10,848,224	9,562.34	130	london
**Term**	241,896,559	492.37	1	rt
**Feature usage by #Users**				
**Hashtag**	592,363	10.08	1	#retweet
**Mention**	26,293	5.44	1	dimensionist
**Location**	739,120	641.5	2	london
**Term**	1,799,385	6,616.65	1	rt
**Feature using #Hashtags**				
**User**	18,167	2	0	daily_astrodata
**Location**	2,440,969	1,837.79	21	uk

**Figure 1:**
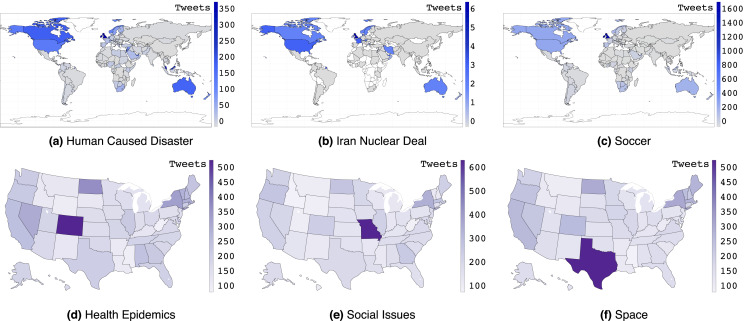
(A–F) *Per capita* tweet frequency across different international and U.S. locations for different topics. The legend provides the number of tweets per 1 million capita.

Figure 1 shows *per capita* tweet frequency across different international and U.S. locations for different topics. While English speaking countries dominate English tweets, we see that the Middle East and Malaysia additionally stand out for the topic of Human Caused Disaster (MH370 incident), Iran, U.S., and Europe for nuclear negotiations the “Iran Nuclear deal”, and soccer for some (English-speaking) countries where it is popular. For U.S. states, we see that Colorado stands out for health epidemics (both whooping cough and pneumonic plague), Missouri stands out for social issues (#blacklivesmatter in St. Louis), and Texas stands out for space due to NASA’s presence there.

## Methodology

In this section, we describe the formal framework we use for our longitudinal study of topic classification. We begin by describing how we automatically label data using a set of manually curated hashtags. Then, we proceed to describe how we temporally split both the dataset and manually curated hashtags into train, validation and test sets, which is a critical step for our longitudinal study of topical classifiers and long-term generalization. Finally, we provide a brief description of several score-based classification algorithms and one ranking algorithm used in our analysis.

### Dataset labelling

A critical bottleneck for learning targeted topical social classifiers is to achieve sufficient supervised content labeling. With data requirements often in the thousands of labels to ensure effective learning and generalization over a large candidate feature space (as found in social media), manual labeling is simply too time-consuming for many users, while crowdsourced labels are both costly and prone to misinterpretation of users’ information needs. Fortuitously, hashtags have emerged in recent years as a pervasive topical proxy on social media sites—hashtags originated on Internet Relay Chat (IRC), were adopted later (and perhaps most famously) on Twitter, and now appear on other social media platforms such as Instagram, Tumblr, and Facebook. Following the approach of Lin, Snow & Morgan (2011), for each topic *t* ∈ *T*, we leverage a (small) set of user hand-curated topical hashtags *H*^*t*^ to efficiently label a large number of supervised topic labels for social media content.

Specifically, we manually curated a broad thematic range of 10 topics shown in the top row of Table 2 by annotating hashtag sets *H*^*t*^ for each topic *t* ∈ *T*. We used four independent annotators to query the Twitter search API to identify candidate hashtags for each topic, requiring an inter-annotator agreement of three annotators to permit a hashtag to be assigned to a topic set. Samples of hashtags for each topic are given in the bottom row of Table 2.

**Table 2:** Train/Validation/Test Hashtag samples and statistics.

	Tennis	Space	Soccer	Iran nuclear deal	Human disaster	Celebrity death	Social issues	Natural disaster	Epidemics	LGBT
**#TrainHashtags**	62	112	144	12	57	33	37	61	55	30
**#ValHashtags**	14	32	42	2	8	4	5	4	17	9
**#TestHashtags**	14	17	21	3	12	7	8	17	13	5
**#+TrainTweets**	21,716	5,333	14,006	6,077	153,612	155,121	27,423	46,432	14,177	1,344
**#-TrainTweets**	191,905	46,587	123,073	54,045	1,363,260	1,376,872	244,106	411,609	125,092	11,915
**#+ValTweets**	884	2,281	4,073	1,261	53,340	23,710	3,088	843	4,348	50
**#-ValTweets**	7,860	20,368	36,341	11,363	473,791	210,484	27,598	7,456	39,042	443
**#+TestTweets**	1,510	5,908	11,503	368	34,055	7,334	14,566	5,240	3,105	692
**#-TestTweets**	13,746	53,348	103,496	3,256	305,662	65,615	130,118	47,208	27,828	6,325
**Sample Hashtags**	#usopenchampion	#asteroids	#worldcup	#irandeal	#gazaunderattack	#robinwilliams	#policebrutality	#earthquake	#ebola	#loveislove
	#novakdjokovic	#astronauts	#lovesoccer	#iranfreedom	#childrenofsyria	#ripmandela	#michaelbrown	#storm	#virus	#gaypride
	#wimbledon	#satellite	#fifa	#irantalk	#iraqwar	#ripjoanrivers	#justice4all	#tsunami	#vaccine	#uniteblue
	#womenstennis	#spacecraft	#realmadrid	#rouhani	#bombthreat	#mandela	#freetheweed	#abfloods	#chickenpox	#homo
	#tennisnews	#telescope	#beckham	#nuclearpower	#isis	#paulwalker	#newnjgunlaw	#hurricanekatrina	#theplague	#gaymarriage

### Dataset splitting

In the following, we describe key aspects related to the temporal splitting of the dataset and *H*^*t*^ labels for training, validation parameter tuning, and test evaluation purposes. We also outline a methodology for sampling negative examples and an overall training procedure including hyperparameter tuning.

**Temporal splits of data and *H***^***t***^ **for training, validation and testing: **As standard for machine learning methods, we divide our training data into train, validation, and test sets—the validation set is used for hyperparameter tuning to control overfitting and ensure generalization to unseen data. As a critical insight for topical generalization where we view correct classification of tweets with *previously unseen topical hashtags* as a proxy for topical generalization, we do not simply split our data temporally into train and test sets and label both with *all* hashtags in *H*^*t*^. Rather, we split each *H*^*t*^ into three disjoint sets $H^t_{\rm train}$, $H^t_{\rm val}$, and $H^t_{\rm test}$ according to two time stamps $t^{\rm train}_{\rm split}$ and $t^{\rm val}_{\rm split}$ for topic *t* and the first usage time stamp *h*_time_* of each hashtag *h* ∈ *H*^*t*^. In short, all hashtags *h* ∈ *H*^*t*^ first used before $t^{\rm train}_{\rm split}$ are used to generate positive labels in the training data, all hashtags *h* ∈ *H*^*t*^ first used after $t^{\rm train}_{\rm split}$ and before $t^{\rm val}_{\rm split}$ are used to generate positive labels in the validation data, and the remaining hashtags are used to generate positive labels in the test data. Here we first outline the procedure and follow later with a detailed explanation.

To achieve this effect formally, we define the following:

$H_{{\rm train}}^t = \{ h|h \in {H^t} \wedge {h_{{\rm time*}}} < t_{{\rm split}}^{{\rm train}}\}$

$H_{{\rm val}}^t = \{ h|h \in {H^t} \wedge {h_{{\rm time*}}} \ge t_{{\rm split}}^{{\rm train}} \wedge {h_{{\rm time*}}} < t_{{\rm split}}^{{\rm val}}\}$

$H_{{\rm test}}^t = \{ h|h \in {H^t} \wedge {h_{{\rm time*}}} \ge t_{{\rm split}}^{{\rm val}}\}$

Once we have split our hashtags into training and validation sets according to $t_{{\rm split}}^{{\rm train}}$ and $t_{{\rm split}}^{{\rm val}}$, we next proceed to temporally split our training documents *D* into a training set $D_{{\rm train}}^t$, a validation set $D_{{\rm val}}^t$, and a test set $D_{{\rm test}}^t$ for topic *t* based on the posting time stamp *d*_*i*,time_* of each tweet *d*_*i*_ as follows:

$D_{{\rm train}}^t = \{ {d_i}|{d_i} \in D \wedge {d_{i,{\rm time*}}} < t_{{\rm split}}^{{\rm train}}\}$

$D_{{\rm val}}^t = \{ {d_i}|{d_i} \in D \wedge {d_{i,{\rm time*}}} \ge t_{{\rm split}}^{{\rm train}} \wedge {d_{i,{\rm time*}}} < t_{{\rm split}}^{{\rm val}} \wedge (\forall h \in {d_i}:h\ \notin\ H_{{\rm train}}^t)\}$

$D_{{\rm test}}^t = \{ {d_i}|{d_i} \in D \wedge {d_{i,{\rm time*}}} \ge t_{{\rm val}}^{{\rm train}} \wedge (\forall h \in {d_i}:h\ \notin\ H_{{\rm train}}^t)\}$

Finally, to label the train, validation, and test data sets $D_{{\rm train}}^t$, $D_{{\rm val}}^t$ and $D_{{\rm test}}^t$, we use the *respective* hashtag sets $H_{{\rm train}}^t$, $H_{{\rm val}}^t$, $H_{{\rm test}}^t$ for generating the topic label for a particular tweet *t*(*d*_*i*_) ∈ {0, 1} as follows, where we take a set-based view of the features positively contained in vector *d*_*i*_:

$t({d_i}) = \left\{ {\matrix{ 1 & {{\rm if \;}{d_i} \in D_{{\rm train}}^t \wedge \exists \;h \in {d_i}:h \in H_{{\rm train}}^t} \cr 1 & {{\rm if \;}{d_i} \in D_{{\rm val}}^t \wedge \exists \;h \in {d_i}:h \in H_{{\rm val}}^t} \cr 1 & {{\rm if \;}{d_i} \in D_{{\rm test}}^t \wedge \exists \;h \in {d_i}:h \in H_{{\rm test}}^t} \cr 0 &{{\rm otherwise}} \cr } } \right..$

The critical insight here is that we not only divide the train, validation, and test data temporally, but we also divide the hashtag labels temporally and label the validation and test data with an entirely disjoint set of topical labels from the training data. The purpose behind this training, validation and test data split and labeling is to ensure that hyperparameters are tuned so as to prevent overfitting and maximize generalization to unseen topical content (*i.e*., new hashtags). We remark that by doing this, *a classifier that simply memorizes training hashtags will fail to correctly classify the validation data* except in cases where a tweet contains both a training and validation hashtag. Moreover, we argue that removing tweets containing training hashtags from the validation data is important since ranking these tweets highly does not provide any indication of classifier generalization beyond the training hashtags. We later experimentally validate this tweet removal proposal against a baseline where (a) we include all train hashtags $H_{{\rm train}}^t$ in the validation hashtag set $H_{{\rm val}}^t$ and (b) we include all tweets *d*_*i*_ containing these train hashtags in the validation dataset $D_{{\rm val}}^t$.

Per topic, hashtags were split into train and test sets according to their first usage time stamp roughly according to a 3/5 to 2/5 proportion (the test interval spanned between 9–14 months). The train set was further temporally subdivided into train and validation hashtag sets according to a 5/6 to 1/6 proportion. We show a variety of statistics and five sample hashtags per topic in Table 2. Here we can see that different topics had varying prevalence in the data with *Soccer* being the most tweeted topic and *Iran Nuclear Deal* being the least tweeted according to our curated hashtags.

**Sampling negative examples:** Topic classification is often considered to be an imbalanced classification task since usually there are many more negative examples than positive examples. Indeed, the large number of users on Twitter, their diversity, their wide range interests, and the short lifetime of topics discussed on a daily basis typically imply that each topic has only a small set of positive examples. For example, in the “natural disaster” topic that we evaluate in this article, we remark that we have over 800 million negative examples and only 500,000 positive examples. Therefore, given this extreme class imbalance, we have chosen to subsample negative examples, which is commonly used to enable faster training and more effective hyperparameter tuning compared to training with all negative examples. Specifically, we randomly subsample negative examples such that positive examples represent 10% of the dataset for each topic while negative examples represent 90% of the dataset. This rule is valid for the training, validation and test sets of each topic. Detailed statistics of each topic dataset are provided in Table 2.

**Training and hyper-parameter tuning:** Once $D_{{\rm train}}^t$ and $D_{{\rm val}}^t$ have been constructed, we proceed to train our scoring function *f*^*t*^ on $D_{{\rm train}}^t$ and select hyperparameters to optimize Average Precision (AP)^2^ 2See Manning, Raghavan & Schütze (2008) for a discussion and definition of this commonly used ranking metric. on $D_{{\rm val}}^t$. Once the optimal *f*^*t*^ is found for $D_{{\rm val}}^t$, we return it as our final learned topical scoring function *f*^*t*^ for topic *t*. Because ${f^t}({d_i}) \in {\rm {\mathbb R}}$ is a scoring function, it can be used to rank.

With train, validation, and testing data defined along with the training methodology, it remains now to extract relevant features, described next.

### Topic classification features

The set of features that we consider for each tweet *d*_*i*_ are: (i) *User* (author of the tweet), (ii) *Mention*, (iii) *Location*, (iv) *Term*, and (v) *Hashtag* features. Because we have a total of 538,365,507 unique features in our Twitter *corpus* (the total count of unique feature values is shown in Table 1), it is critical to pare this down to a size amenable for efficient learning and robust to overfitting. To this end, we thresholded all features according to the frequencies listed in Table 3. The rationale for our frequency thresholding was to have roughly 1 million features with equal numbers of each feature type. We also removed common English stopwords which further reduced the unique term count. Overall, we end up with 1,017,935 candidate features (*CF*) for learning topical classifiers.

**Table 3:** Cutoff threshold and corresponding number of unique values of candidate features *CF* for learning. Thresholds were chosen to balance the number of each type of feature.

	Frequency threshold	#Unique values
**User**	235	206,084
**Hashtag**	65	201,204
**Mention**	230	200,051
**Location**	160	205,884
**Term**	200	204,712
**Total candidate**		
**Features (*CF*)**	–	1,017,935

### Supervised learning algorithms

With our labeled training, validation, and test datasets and our candidate feature set *CF* now defined, we proceed to apply different probabilistic classification and ranking algorithms to generate a scoring function *f*^*t*^ for learning topical classifiers as defined previously. In this article, we experiment with the following five state-of-the-art supervised classification and ranking methods:

1. **Logistic Regression (LR)** (Fan et al., 2008): LR uses a logistic function to predict the probability that a tweet is topical. We used *L*_2_ regularization with the hyperparameter *C* (the inverse of regularization strength) selected from a search over the values *C* ∈ {10^−12^, 10^−11^, …, 10^11^, 10^12^}.
2. **Naïve Bayes (NB)** (McCallum & Nigam, 1998): NB makes a naïve assumption that all are features are independent conditioned on the class label. Despite the general incorrectness of this independence assumption, McCallum & Nigam (1998) remark that it is known to make an effective topic classifier. Like LR, NB predicts the probability that a tweet is topical. For parameter estimation, we used Bayesian smoothing using Dirichlet priors with hyperparameter *α* selected from a search over the values *α* ∈ {10^−20^, 10^−15^, 10^−8^, 10^−3^, 10^−1^, 1}.
3. **RankSVM** (Lee & Lin, 2014): RankSVM is a variant of the support vector machine algorithm used to learn from pairwise comparison data (in our case pairs consist of a positive labeled datum that should be ranked above a negatively labeled datum) that naturally produces a ranking. We used a linear kernel with the regularization hyperparameter *C* (the trade-off between training error and margin) selected in the range *C* ∈ {10^−12^, 10^−11^, …, 10^11^, 10^12^}.
4. **Random Forest (RF)** (Breiman, 2001): RF is an ensemble learning method for classification that operates by constructing a multitude of decision trees at training time and predicting the class that is the mode of the class prediction of the individual trees (the number of trees that predict the most common class being the score). RF is known to be a classifier that generalizes well due to its robustness to overfitting. For RF, we tuned the hyperparameter for the number of trees in the forest selected from a search over the respective values {10, 20, 50, 100, 200}.
5. **k-Nearest Neighbors (k-NN)** (Aha, Kibler & Albert, 1991): k-NN is a non-parametric method used for classification. An instance is classified by a plurality vote of its *k* neighbors, with the object being assigned to the class most common among its *k* nearest neighbors (the number of *k* neighbors for the most common class being the score). The value of *k* is the primary hyperparameter for k-NN and was selected from a search over the respective values {1, 2, 3, …, 10}.

We remark that almost all algorithms performed better with feature selection and hence we used feature selection for all algorithms, where the number of top features *M* was selected in a topic-specific manner based on their Mutual Information with the topic being classified. *M* was tuned over values in {10^2^, 10^3^, 10^4^, 10^5^}. As noted previously, hyperparameter tuning is done *via* exhaustive grid search using the Average Precision (AP) ranking metric on validation data. All code to process the raw Twitter data and to train and evaluate these classifiers as described above is provided on github (https://github.com/SocialSensorProject/socialsensor).

In the next section, we present results for an intensive evaluation of these classifiers for our longitudinal study of topic classification on the Twitter data previously described.

## Results and Discussion

We now report and discuss the main results of our longitudinal study of topic classification on Twitter.

### Classification performance analysis

In the following, we first conduct an analysis of the overall classification performance by comparing the classifiers described above, and then, we describe the outcome of a longitudinal classification performance.

#### Overall classification performance

While our training data is provided as supervised class labels, we remark that topical classifiers are targeted towards individual users who will naturally be inclined to *examine only the highest ranked tweets*. Hence we believe ranking metrics represent the best performance measures for the intended use case of this work. While RankSVM naturally produces a ranking, all classifiers are score-based, which also allows them to provide a natural ranking of the test data that we evaluate *via* the following ranking metrics:

- **AP:** Average Precision over the ranked list (Manning, Raghavan & Schütze, 2008); the mean over all topics provides the Mean Average Precision (MAP).
- **P@*k*:** Precision at *k* for *k* ∈ {10, 100, 1000}.

While P@10 may be a more standard retrieval metric for tasks such as ad-hoc web search, we remark that the short length of tweets relative to web documents makes it more plausible to look at a much larger number of tweets, hence the reason for also evaluating P@100 and P@1000.

Table 4 evaluates our chosen ranking metrics for each topic. *Random Forest* is the best performing method on average, except for *P*@1000 where *Logistic Regression* performed *slightly* better in the 3rd significant digit. The generally strong performance of *Random Forest* is due to its robustness to overfitting Breiman (2001). In general, *KNN* is only slightly worse than *Logistic Regression*, while *Naïve Bayes* and *RankSVM* typically perform worse. Notably, trained classifiers outperform *RankSVM* on the ranking task thus justifying the use of trained topic classifiers for ranking.

**Table 4:** Performance of topical classifier learning algorithms across metrics and topics with the mean performance over all topics shown in the right column with *±* 95% confidence intervals. The best mean performance per metric is shown in bold.

		Tennis	Space	Soccer	Iran nuclear deal	Human disaster	Celebrity death	Social issues	Natural disaster	Epidemics	LGBT	Mean
**LR**	**AP**	**0.9590**	0.6452	0.5036	**0.9807**	**0.6952**	0.9293	0.5698	**0.9428**	0.4005	0.1559	0.6782 *±* 0.1724
**NB**	**AP**	0.5859	0.8471	0.3059	0.9584	0.4224	0.4658	0.5030	0.3518	0.4050	0.1689	0.5014 *±* 0.1494
**RankSVM**	**AP**	0.702	0.840	**0.674**	0.586	0.603	0.469	0.370	0.248	0.136	0.082	0.471 *±* 0.18
**RF**	**AP**	0.9344	**0.9314**	0.5509	0.9757	0.6658	**0.9571**	**0.8213**	0.8306	**0.5154**	**0.2633**	**0.7445 *±* 0.14764**
**KNN**	**AP**	0.9550	0.7751	0.4739	0.9752	0.598	0.542	0.5078	0.9599	0.5317	0.1774	0.6496 *±* 0.1618
**LR**	**P@10**	**1.0**	0.2	0.3	**1.0**	0.5	0.8	0.2	**1.0**	0.5	**0.6**	0.61 *±* 0.2012
**NB**	**P@10**	0.1	**0.8**	0.0	0.9	0.7	0.1	0.0	0.3	0.1	0.0	0.3 *±* 0.2225
**RankSVM**	**P@10**	**1.0**	**0.8**	**0.6**	0.8	0.4	0.3	0.0	0.1	0.0	0.2	0.42 *±* 0.26
**RF**	**P@10**	**1.0**	0.5	0.5	**1.0**	**0.9**	**1.0**	**1.0**	**1.0**	**0.7**	0.5	**0.81 *±* 0.1444**
**KNN**	**P@10**	**1.0**	0.0	1.0	**1.0**	0.7	0.9	0.0	0.9	0.3	0.4	0.62 *±* 0.2543
**LR**	**P@100**	**0.98**	0.65	**0.44**	**0.99**	0.74	0.94	0.59	**0.98**	0.45	0.2	0.696 *±* 0.1721
**NB**	**P@100**	0.56	**0.95**	0.0	0.98	0.39	0.36	0.16	0.37	0.48	0.1	0.435 *±* 0.2033
**RankSVM**	**P@100**	0.73	0.72	0.31	0.70	**0.88**	0.44	0.48	0.34	0.02	0.100	0.472 *±* 0.20
**RF**	**P@100**	**0.98**	0.94	0.43	0.98	0.62	**0.97**	**0.81**	0.9	**0.61**	**0.29**	**0.753 *±* 0.1555**
**KNN**	**P@100**	1.0	0.59	0.34	1.0	0.72	0.54	0.39	0.96	0.54	0.24	0.632 *±* 0.1731
**LR**	**P@1000**	0.653	0.703	0.545	0.299	**0.666**	0.884	0.574	0.919	0.267	0.076	**0.5586 *±* 0.1682**
**NB**	**P@1000**	0.551	0.667	0.29	**0.333**	0.338	0.542	0.655	0.287	0.319	**0.169**	0.4151 *±* 0.1073
**RankSVM**	**P@1000**	**0.799**	**0.922**	**0.764**	0.218	0.525	0.547	0.215	0.173	0.154	0.064	0.438 *±* 0.22
**RF**	**P@1000**	0.728	0.464	0.576	0.331	0.463	**0.914**	**0.789**	0.728	**0.397**	0.159	0.5549 *±* 0.145
**KNN**	**P@1000**	0.571	0.821	0.53	0.329	0.476	0.84	0.49	**0.929**	0.234	0.083	0.5303 *±* 0.1696

To provide more insight into the general performance of our learning topical classifier framework, we provide the top five tweets for each topic according to *Logistic Regression*^3^ 3*Logistic Regression* allows us to better understand failure cases for topical classifiers, *i.e*., *Random Forest* is likely to have gotten all of the top-5 right. in Table 5. We have annotated tweets with symbols as follows:

**Table 5:** Top tweets for each topic from *Logistic Regression* method results, marked with ✗ as irrelevant, ✓ as relevant and labeled as topical, and ★ as relevant but labeled as non-topical (a mislabeled example).

**Tennis**	**Space**
✓ PHOTOS; @andy_murray in @usopen QF match v Novak Djokovic … @usta @BritishTennis #USOpen2014…	✗ RT @wandakki: Chuck’s Story - My 600-lb Life — http://t.co/aP3L1OqIch — Reality TV #tv #episode #Reality #TV…
✓ PHOTOS; British #1 @andy_murray in @usopen Quarter-Finals match v Novak Djokovic … @usta @BritishTennis #USOpen2014…	✗ RT @arist_brain: Path. #Switzerland (by Roman Burri) #travel #landscape #nature #path #sky #alps #clouds…
✓ RT @fi_sonic: PHOTOS; @andy_murray in 75 75 64 win over Jo-wilfried Tsonga to reach @usopen QFs. @BritishTennis…	✗ TeamFest Winner Circle by Dee n Ralph on Etsy--Pinned with http://t.co/Cr1PC31naR #beach #ocean #sea #love…
✓ PHOTOS; #21 seed @sloanetweets in her @usopen 2nd round match v Johanna Larsson … @USTA @WTA #USOpen2014…	✓ RT @NASA: Fire @YosemiteNPS as seen by NASA’s Aqua satellite on Sunday. #EarthRightNow…
✓ “ @fi_sonic: PHOTOS; @DjokerNole celebrating his @usopen QF match win 76 67 62 64 v Andy Murray … @usta #USOpen2014…	✓ RT @NASA: Arkansas April 27 tornado track seen by NASA’s EO-1 satellite. http://t.co/d36sKPGzAx #EarthRightNow…
**Soccer**	**Iran Nuclear Deal**
✓ RT @FOXSoccer: Cameron in for Beckerman #USA lineup: Howard, Gonzalez, Bradley, Besler, Beasley, Dempsey…	✓ RT @JavadDabiran: #Iran-Executions, #Women rights abuse, #IranHRviolations soar under Hassan Rouhani #No2Rouhani…
✓ RT @FOXSoccer: Cameron in for Beckerman #USA lineup: Howard, Gonzalez, Bradley, Besler, Beasley, Dempsey	✓ RT @HellenaRezai: #Iran-Executions, #Women rights abuse, #IranHRviolations soar under Hassan Rouhani #No2Rouhani…
★ RT @Gerrard8FanPage: Luis Suarez has scored seven goals in six Barclays Premier League appearances against Sunderland.	✓ RT @peymaneh123: #Iran-Executions, #Women rights abuse, #IranHRviolations soar under Hassan Rouhani #No2Rouhani…
★ RT @BBCMOTD: Federico Fazio is the first player sent off on his PL debut since Samba Diakite for #QPR in Feb 2012 #THFC…	✓ RT @IACNT: #Iran nuclear threat bigger than claimed: http://t.co/13Qk7cyWyA @SenTedCruz @JohnCornyn #nuclear…
★ @JamesYouCun* well I’d say Migs, moreno sakho toure (if fit) manquilio Lucas can gerrard sterling Coutinho markovic and borini	✓ RT @YelloJackets: #Iran-Executions, Women rights abuse and #IranHRviolations soar under Hassan Rouhani
**Human Disaster**	**Celebrity Death**
✓ @IlenePrusher if one thinks of Gazan kids as potential Hamas fighters Gazan women as potential Hamas fighters’ mothers, yes!	✓ #RIPRise Heaven gained another angel yet another angel, you will be happy with EunB, all our prayers are for you…
★ RT @jala_leb: This is GAZA not Hiroshima @BarackObama @David_Cameron @un @hrw http://t.co/ddZWORPqrQ	✓ RT @WeGotLoves: EunB, Manager, Driver Rise passed away. Very heartbreaking news. Deep condolences to their family.
✓ RT @jallubk: THIS AGAIN: BOYCOTT ISRAEL OR WE WILL BOYCOTT YOU, @robbiewilliams ! #IsraelKillsKids…	✓ RT @sehuntella: eunb, manager, driver and rise passed away. what a heartbreaking news. deep condolences to their family
✗ RT @notdramadriven: Nailed it @KenWahl1 @DrMartyFox @jjauthor @shootingfurfun @CarmineZozzora…	✓ RT @missA_TH: Our deep condolences to family, friends and fans of EunB Rise. May they rest in peace. Heaven has
✓ RT @TelecomixCanada: @Op_Israel #Article51 of the Geneva Convention: http://t.co/VaDklflx5C Tick Tock	✓ Rest in peace Rise! Heaven now gained two angels. #RipRise #PrayForLadiesCode My condolences :(
**Social Issues**	**Natural Disaster**
✓ RT @RightCandidates: THANK YOU DEMOCRAT RACE BAITERS #tcot #america #women #millennials #tlot…	✓ RT @ianuragthakur: I appeal to friends supporters @BJYM to help in the relief efforts fr #KashmirFloods…
✗ RT @2AFight: The Bill of Rights IS my Patriot Act #2A #NRA #MolonLabe #RKBA #ORPUW #PJNET #tgdn…	✓ RT @RSS_Org: RSS Press Release: An Appeal to the Society to donate for Relief Fund to help #KashmirFloods Victims…
✗ The Supreme Court Judicial Tyranny http://t.co/HKo4hnQnF5 #1A #MakeDCListen #NObamaCARE#KeystoneXL…	✓ RT @punkboyinsf: #BREAKING California Gov. Jerry Brown has declared a state of emergency following…
✓ RT @RightCandidates: THANK YOU DEMOCRAT RACE BAITERS FOR THIS #tcot #america #women #FergusonDecision…	✓ RT @nbcbayarea: #BREAKING California Gov. Jerry Brown has declared a state of emergency following…
✗ Race-Baiting for Profit RT http://t.co/KOYfDDNQCu #TCOT #CCOT #MakeDCListen #TeaParty #Conservatives	✓ RT @coolfunnytshirt: Congress ke bure din! RT @timesnow: Congress leader Saifuddin Soz heckled by flood victims
**Epidemics**	**LGBT**
✓ RT @justgrateful: Surgeon General Nominee is Blocked by NRA #occupy #uppers #tcot #ccot #topprog #EbolaCzar…	✗ RT @CSGV: Take a bite out of the crime. Oppose traitors preparing for war w/ our gov’t. #NRA #NRAAM Cliven Bundy…
✓ RT @nhdogmom: Why don’t we have Surgeon General/Medical #EbolaCzar … GOP RWNJ’s is why!!…	✗ IRS employee suspended for pro-Obama… - Washington Times: http://t.co/KoCtwaJ0C6*via* @washtimes Another meaningless…
✗ New York seen like never before! #cool #photo #black white #atmospheric #moody	✓ Pa. gay-marriage ban overturned http://t.co/Gl4kAhQwyQ*via* @phillydotcom #lovewins #lgbt
✗ RT @ryangrannand:.@CouncilW9 asking developer for a sign plan. #waltham	✓ RT @OR4Marriage: RT this AMAZING quote from yesterday’s ruling striking down #Oregon’s marriage ban! #OR4M #lgbt…
✗ GOOD OFFER!! http://t.co/1qm1K0UIaw Vitamins Supplements, Clinically Proven-Doctor Formulated	✓ @briansbrown YOU ANTI-GAY BIGOTS ARE BOX-OFFICE-POISON EVEN FOR MOST REPUBLICANS. #LGBT…

- ✓: the tweet was labeled topical by our test hashtag set.
- ★: the tweet was determined to be topical through manual evaluation even though it did not contain a hashtag in our curated hashtag set (*this corresponds to a mislabeled example due to the non-exhaustive strategy used to label the data*).
- ✗: the tweet was not topical.

In general, we remark that our topical classifier may perform slightly better than the quantitative results in Table 4 would indicate: a few of the highly ranked tweets are mislabeled as non-topical in the test set although a manual analysis reveals that they are in fact topical. Furthermore, even though we use hashtags to label our training, validation, and testing data, our topical classifier has highly (and correctly) ranked topical tweets that *do not contain hashtags*, indicating strong generalization properties from a relatively small set of curated topical hashtags.

Though the reason why some non-topical tweets ranked highly is unclear, we see that many failure cases appear to mention relevant features to the topic although they are in fact advertising or politicized spam content. This indicates a limitation of the hashtag-based class labeling method, which cannot easily distinguish spam from legitimate content. Nonetheless, we believe that a separate spam filter common to all classifiers could mitigate these issues since the patterns of spam email such as an unusually large number of hashtags or mentions are not topic-specific and can be easily detected.

#### Longitudinal classification performance

Now that we’ve examined the overall classification performance of different topical classifiers per topic and per metric, we now turn to address the long-term temporal aspect of topic classification with two questions: (1) Does classification performance degrade as time increases since training, and if so, by how much? (2) Does omission of training hashtags from the validation set encourage better long-term generalization since, as hypothesized in the methodology, it discourages memorizing training hashtags?

**Figure 2:**
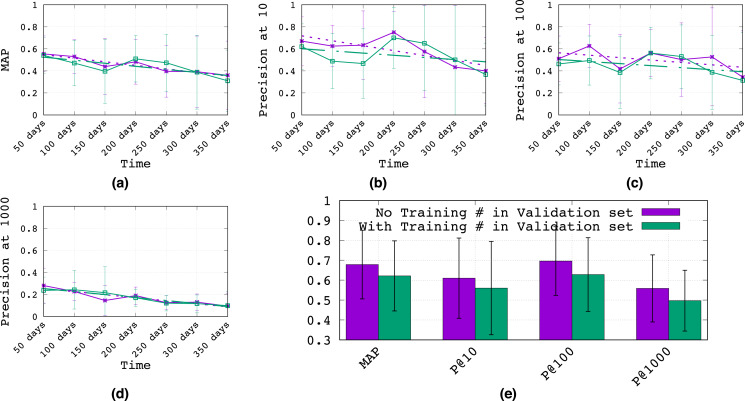
Longitudinal analysis of classifier generalization. (A–D) The performance of the topic classifier (mean over all 10 topics with 95% confidence intervals) from 50 to 350 days after training, evaluated according to (A) mean AP (MAP), (B) P@10, (C) P@100, and (D) P@1000. Best fit linear regressions are shown as dashed lines. (E) Results averaged over time with 95% confidence intervals.

To assess these questions, Figs. 2A–2D plots the performance of the *Logistic Regression*^4^ 4We could not run these longitudinal experiments with *Random Forest* due to the significant computational expense of the analysis in this section and the hyperparameter tuning that is required, thus we opted to perform this analysis with the much faster and still strongly competitive *Logistic Regression* classifier. topic classifier (mean over all 10 topics) from 50 to 350 days after training, evaluated according to (a) mean AP (MAP), (b) P@10, (c) P@100, and (d) P@1000. The purple line shows the proposed methodology, where tweets with training hashtags are suppressed from the validation set, while the green line does not suppress training hashtags (see the Methodology section for more details on both methods). To better distinguish the overall performance of suppressing training hashtags in the validation set, we average results over all time points in Fig. 2E.

Overall, we make a few key observations:

- Regarding question (1), it is clear that the classification performance drops over time–a roughly 35% drop in MAP from the 50th to the 350th day after training. Clearly, there will be topical drift over time for most topics (*e.g*., Natural Disasters, Social Issues, Epidemics) as different events occur and shift the focus of topical conversation. While there are more sophisticated training methods for mitigating some of this temporal drift (*e.g*., Wang et al., 2019), overall, it would seem that the most practical and effective method for long-term generalization would involve a periodic update of training hashtags and data labels.
- Regarding question (2), Fig. 2E clearly shows an overall performance improvement from discarding training hashtags (and their tweets) from the validation set. In fact, for MAP alone, we see roughly an 11% improvement. Hence, these experiments suggest there may be a long-term generalization advantage to excluding training hashtags from the validation hashtags and data, which we conjecture discourages hyperparameters that lead to hashtag memorization from the training set.

With our comparative and longitudinal analysis of topic classifier performance now complete, we will next investigate which features are most informative for topic classifiers.

### Feature analysis

In this section, we analyze the informativeness of feature sets defined in the Data Description section and the effect of their attributes on learning targeted topical classifiers. To this end, our goal in this section is to answer the following questions:

- What are the best features for learning classifiers and do they differ by topic?
- For each feature type, do any attributes correlate with importance?

To answer these questions, we use Mutual Information (MI) (Manning, Raghavan & Schütze, 2008) as our primary metric for feature evaluation. MI is a general method for measuring the amount of information one random variable contains about another random variable and is used to select predictive features in machine learning. To calculate the amount of information that each feature *j* in the Candidate Features (*CF*) defined previously provides w.r.t. each topic label *t* ∈ {Natural Disaster, Epidemics, …}, MI is formally defined as

$I(j,t) = \sum\limits_{t \in \{ {\rm 0},{\rm 1}\} } \sum\limits_{j \in \{ {\rm 0},{\rm 1}\} } p(j,t)\log \left( {\displaystyle{{p(j,t)} \over {p(j)p(t)}}} \right)$

with marginal probabilities of topic *p*(*t*) and feature *p*(*j*) occurrence and joint probability *p*(*t*, *j*) computed empirically over the sample space of all tweets, where higher values for this metric indicate more informative features *j* for the topic *t*.

**Figure 3:**
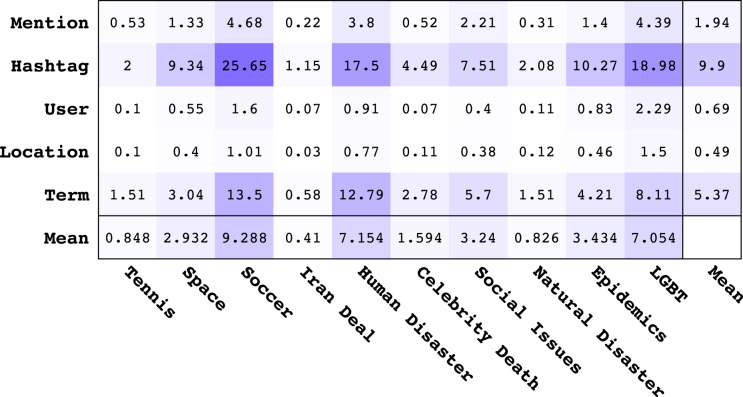
Matrix of mean Mutual Information values for different feature types *vs*. topics. The last column and last row represent the average of mean values across all topics and all features respectively. All values should be multiplied by 10^−8^.

**Figure 4:**
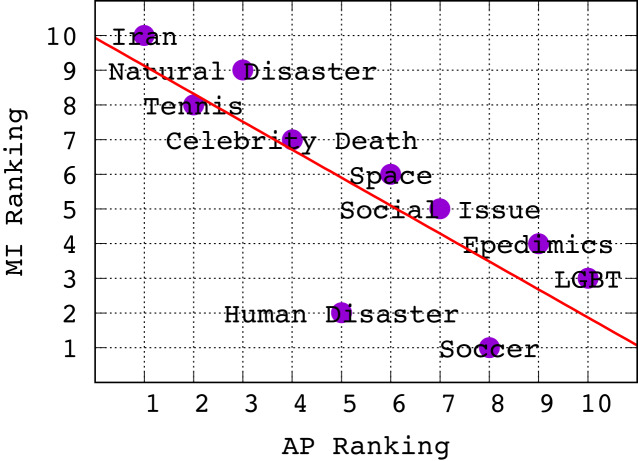
Scatter plot showing ranking of topics w.r.t. Mutual Information *vs*. Average Precision. There is clearly a negative correlation, with a Kendall τ coefficient of −0.68.

In order to assess the overall best feature types for learning topical classifiers, we provide the mean MI values for each feature type across different topics in Fig. 3. The last column in Fig. 3 shows the average of the mean MI for each feature type and the last row shows the average of the mean MI for each topic. From analysis of Fig. 3, we make the following observations:

- Looking at the average MI values, the order of informativeness of feature types is the following: *Hashtag*, *Term*, *Mention*, *User*, *Location*. The overall informativeness of *Hashtags* is not surprising given that hashtags are used on Twitter to tag topics of interest. While the *Term* feature is not strictly topical, it contains a rich vocabulary for describing topics that *Mention*, *User*, and *Location* lack.
- The *Location* feature provides high MI regarding the topics of *Human Disaster*, *LBGT*, and *Soccer* indicating that a lot of content in these topics is geographically localized.
- Revisiting Table 4, we note the following ranking of topics from highest to lowest AP for *Logistic Regression*^5^ 5The ranking for *Random Forest* only differs slightly.: *Iran, Tennis, Natural Disaster, Celebrity Death, Human Disaster, Space, Social Issue, Soccer, Epidemics, LGBT*. It turns out that this ranking is *anti-correlated* with the ranking of topics according to average MI of features in Fig. 3. To establish this relationship more clearly, in Fig. 4 we show a scatterplot of topics according to MI rank *vs*. AP rank. Clearly, we observe that there is a negative correlation between the topic ranking based on AP and MI; in fact, the Kendall *τ* rank correlation coefficient is −0.68 indicating a fairly strong inverse ranking relationship. To explain this, we conjecture that lower average MI indicates that there are fewer good features for a topic; however, this means that classifiers for these topics can often achieve high ranking precision because there are fewer good features and the tweets with those features can be easily identified and ranked highly, leading to high AP. The inverse argument should also hold.

**Figure 5:**
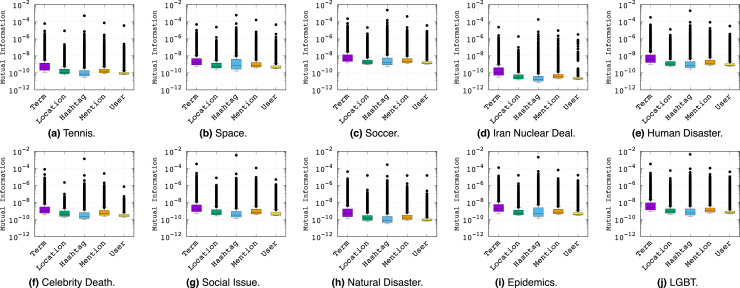
Box plots of Mutual Information values (y-axis) per feature type across topics (x-axis labels).

To further analyze the relationship between the informativeness of feature types and topics, we refer to the box plots of Fig. 5. Here we see the quartiles and outliers of the distribution rather than just the average of the MI values in order to ensure the mean MI values were not misleading our interpretations. Overall, the story of feature informativeness becomes much more complex, with key observations as follows:

- The topic has little impact on which feature is most important, indicating stability of feature type informativeness over topics.
- While *Hashtag* had a higher mean MI score than *Term* in the previous analysis, we see that *Term* has the highest median MI score across all topics, indicating that the high mean MI of *Hashtag* is mainly due to its outliers. In short, the few good *Hashtag* outliers are the overall best individual features, while *Term* has a greater variety of strong (but not absolute best) features.
- Across all topics, *User* is often least informative. However, the distribution of *Location* and *Mention* typically performs competitively with *Hashtag*, although their outliers do not approach the best *Hashtag* features, explaining why *Hashtag* has an overall higher average in Fig. 3.

**Figure 6:**
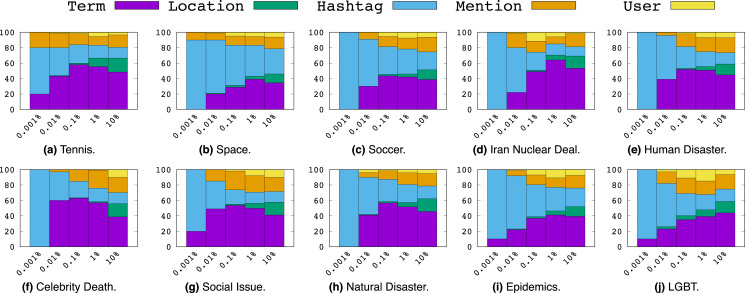
Top *p*% features ranked by Mutual Information.

Now we proceed to a more nuanced analysis of feature types for each topic according to the proportions of their presence among the top *p*% percentiles of MI values for *p*% ∈ {0.001%, 0.01%, 0.1%, 1%, 10%} as shown in Fig. 6. Here we make a few key observations:

- Overall, *Hashtag*s dominate the top 0.001 percentile of features indicating that they account for the most informative features overall.
- However, from percentiles 0.01 to 10, we largely see an increasing proportion of *Term* features among each percentile. This indicates that while the most informative features are *Hashtag*s, there are relatively few of them compared to the number of high MI terms.
- Not to the same extent as *Term*s, we note that *Mention*s also start to become notably more present as the percentile range increases, while *Location*s and *User*s appear least informative overall among the 10th percentile and smaller.

As anecdotal evidence to inspect which features are most informative, we refer to Table 6, which displays the top five feature instances according to MI for each feature type and topic. For example the term *typhoon* is the highest MI term feature with the topic *Natural Disaster*, the official *UNICEF*^6^ 6The United Nations Children’s Fund (UNICEF) is an organization that aims to provide emergency food and healthcare to children and mothers in developing countries everywhere. Twitter account (*@unicef*) is the highest MI feature mention with the Human Disaster topic, and *#worldcup* is (unsurprisingly) the highest MI hashtag feature for the topic *Soccer*. The top *locations are also highly relevant to most topics* indicating the overall importance of these tweet features for identifying topical tweets; for example, three variations of St. Louis, Missouri appear as top MI locations for topic *Social Issues*^7^ 7We remark that the original Black Lives Matter protests originated in St. Louis, Missouri in the aftermath of the police shooting of Michael Brown on August 9, 2014.. One general observation is that *Hashtag* and *Term* features are appear to be the most generic (and hence most generalizable) features, providing strong intuition as to why these features figure so prominently in terms of their informativeness^8^ 8It should also be remarked that Mutual Information (MI) is very sensitive to frequency so a high MI feature must be both informative and frequent to rank highly. This explains why the high MI features are so generic, *i.e*., they are frequent and hence cover many more tweets than low MI features..

**Table 6:** The top five features for each feature type and topic based on Mutual Information.

Topics/Top10	Natural disaster	Epidemics	Iran deal	Social issues	LBGT	Human disaster	Celebrity death	Space	Tennis	Soccer
**User**	from_japan	changedecopine	mazandara	debtadvisoruk	stevendickinson	witfp	boiknox	daily_astrodata	tracktennisnews	makeupbella
**User**	everyearthquake	stylishoz	freeiran9292	nsingerdebtpaid	mgdauber	ydumozyf	jacanews	freesolarleads	novakdjokovic_i	sport_agent
**User**	quakestoday	drdaveanddee	hhadi119	negativeequityf	lileensvf1	syriatweeten	ewnreporter	sciencewatchout	i_roger_federer	yasmingoode
**User**	equakea	soliant_schools	balouchn2	iris_messenger	kevinwhipp	rk70534	rowwsupporter	houston_jobs	andymurrayfans1	sportsroadhouse
**User**	davewinfields	msgubot	jeffandsimon	dolphin_ls	petermabraham	gosyrianews	flykiidchris	lenautilus	rafaelnadal_fan	losangelessrh
**Hashtag**	#earthquake	#health	#iran	#ferguson	#tcot	#syria	#rip	#science	#wimbledon	#worldcup
**Hashtag**	#haiyan	#uniteblue	#irantalks	#mikebrown	#pjnet	#gaza	#ripcorymonteith	#sun	#tennis	#lfc
**Hashtag**	#storm	#ebola	#iranian	#ericgarner	#p2	#israel	#riprobinwilliams	#houston	#usopen	#football
**Hashtag**	#PrayForThePhilippines	#healthcare	#rouhani	#blacklivesmatter	#uniteblue	#gazaunderattack	#rippaulwalker	#starwars	#nadal	#worldcup2014
**Hashtag**	#tornado	#fitness	#irantalksvienna	#icantbreathe	#teaparty	#isis	#robinwilliams	#scifi	#wimbledon2014	#sports
**Location**	With everyone	USA	France	St Louis MO	USA	Syria	South Africa	Houston TX	Worldwide	Liverpool
**Location**	Earth	Francophone	Tehran Iran	Washington DC	Bordentown New Jersey	Palestine	Pandaquotescom	Germany	London	Manchester
**Location**	Philippines	United States	Inside of Iran	St Louis	Global Markets	Syrian Arab Republic	Johannesburg South Africa	Houston	The Midlands	London
**Location**	Don’t follow me am i a bot	Gainesville FL USA	Iran	Virginia US	The blue regime of Maryland	Israel	Johannesburg	Rimouski	London UK	Anfield
**Location**	Global planet earth	Boulder Colorado	Washington DC	Saint Louis MO	Lancaster county PA	Washington DC	Cape Town	In a galaxy far far ebay	Wimbledon	Bangil East Java Indonesia
**Mention**	@oxfamgb	@foxtramedia	@ap	@natedrug	@jjauthor	@ifalasteen	@nelsonmandela	@nasa	@wimbledon	@lfc
**Mention**	@gabriele_corno	@obi_obadike	@afp	@deray	@2anow	@drbasselabuward	@realpaulwalker	@philae2014	@usopen	@fifaworldcup
**Mention**	@weatherchannel	@who	@iran_policy	@antoniofrench	@gop	@revolutionsyria	@ddlovato	@maximaxoo	@atpworldtour	@ussoccer
**Mention**	@twcbreaking	@kayla_itsines	@4freedominiran	@bipartisanism	@pjnet_blog	@unicef	@robinwilliams	@esa_rosetta	@andy_murray	@mcfc
**Mention**	@redcross	@canproveit	@orgiac	@theanonmessage	@espuelasvox	@free_media_hub	@historicalpics	@astro_reid	@wta	@realmadriden
**Term**	typhoon	health	nuclear	police	obama	israeli	robin	space	tennis	liverpool
**Term**	philippines	ebola	regime	protesters	gun	israel	williams	solar	murray	cup
**Term**	magnitude	outbreak	iran	officer	america	gaza	walker	moon	djokovic	supporting
**Term**	storm	virus	iranian	cops	obamacare	palestinian	cory	houston	federer	match
**Term**	usgs	acrx	mullahs	protest	gop	killed	paul	star	nadal	goal

**Figure 7:**
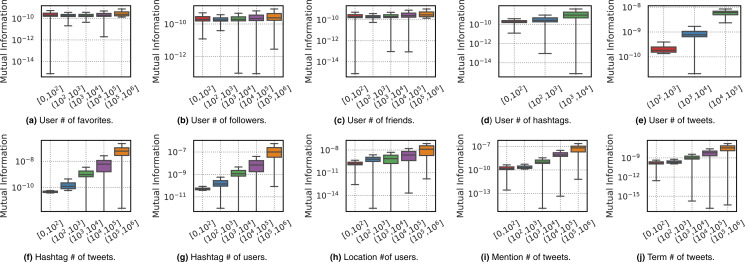
Boxplots for the distribution of Mutual Information values (y-axis) of different features as a function of their attribute values (binned on x-axis). Plots (A–E) respectively show attributes {favorite count, follower count, friend count, hashtag count, tweet count} for *From* feature. Plots (F–J) respectively show attributes tweetCount and userCount for *Hashtag*, userCount for *Location* feature, tweetCount for *Mention* and *Term* features.

In order to answer the second question on whether any attributes correlate with importance for each feature, we provide two types of analysis using the topic *Celebrity Death*–the other topics showed similar patterns, thus we have chosen to omit them. The first analysis shown in Fig. 7 analyzes the distributions of Mutual Information values for features when binned by the magnitude of various attributes of those features, outlined as follows:

- **User***vs*.

– *Favorite count:* # of tweets user has favorited.
– *Followers count:* # of users who follow user.
– *Friends count:* # of users followed by user.
– *Hashtag count:* # of hashtags used by user.
– *Tweet count:* # of tweets from user.

- **Hashtag***vs*.

– *Tweet count:* # of tweets using hashtag.
– *User count:* # of users using hashtag.

- **Location***vs. User count:* # of users using location.
- **Mention***vs. Tweet count:* # of tweets using mention.
- **Term***vs. Tweet count:* # of tweets using term.

As we can see in the boxplots of Fig. 7, the general pattern is that the greater the number of tweets, users, or hashtag count a feature has, the more informative the feature is in general. This pattern also exists to some extent on the attributes of the *From* feature, although the pattern is less visible in general and not clear (or very weak) for the follower or friend count. In general, the informativeness of a user appears to have little correlation with their follower or friend count.

**Figure 8:**
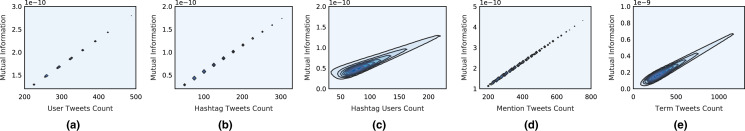
Density plots for the frequency values of feature attributes (x-axis) *vs*. Mutual Information (y-axis). Plots (A–E) respectively show the following attributes: number of tweets for the *User* feature, number of tweets for the *Hashtag* feature, number of users using the *Hashtag* feature, number of tweets for the *Mention* feature, and number of tweets for the *Term* feature.

Figure 8 provides a further analysis by showing density plots of the tweet count attribute of the *User*, *Hashtag*, *Mention* and *Term* features, and the user count attribute of the *Hashtag* feature. Here we can clearly observe the positive linear correlation that exists between the attribute magnitude and the Mutual Information value for all of the evaluated attributes. In short, the more tweets using *User*, *Hashtag*, *Mention* and *Term* features and the more users using a *Hashtag* feature, the more informative that feature typically is for the topic.

## Conclusions

This work provides a long-term study of topic classifiers on Twitter that further justifies classification-based topical filtering approaches while providing detailed insight into the feature properties most critical for topic classifier performance. Our results suggest that these learned topical classifiers generalize well to unseen future topical content over a long time horizon (*i.e*., 1 year) and provide a novel paradigm for the extraction of high-value content from social media. Furthermore, an extensive analysis of features and feature attributes across different topics has revealed key insights including the following two: (i) largely independent of topic, hashtags are the most informative features followed by generic terms, and (ii) the number of unique hashtags and tweets by a user correlates more with their informativeness than their follower or friend count.

Among many interesting directions, future work might evaluate a range of topical classifier extensions: (1) optimizing rankings not only for topicality but also to minimize the lag-time of novel content identification, (2) optimizing queries for boolean retrieval oriented APIs such as Twitter, (3) identification of long-term temporally stable predictive features, (4) utilizing more social network structure as graph-based features, and (5) investigating classifier performance based on topic properties such as periodicity over time or specificity to a very narrow audience. Altogether, we believe these insights will facilitate the continued development of effective topical classifiers for Twitter that learn to identify broad themes of topical information with minimal user interaction and enhance the overall social media user experience.

## Additional Information and Declarations

### Data Availability

The following information was supplied regarding data availability: The code is available at GitHub: https://github.com/SocialSensorProject/socialsensor.
